# Risk factors and prognosis of perioperative acute heart failure in elderly patients with hip fracture: case-control studies and cohort study

**DOI:** 10.1186/s12891-024-07255-x

**Published:** 2024-02-15

**Authors:** Wei Zhao, Mingming Fu, Zhiqian Wang, Zhiyong Hou

**Affiliations:** 1 Department of Geriatric Orthopedics, The Third Hospital of Hebei Medical University, No.139 ZiQiang Lu, Shijiazhuang, 050051 Hebei PR China; 2 Department of Orthopaedic Surgery, The Third Hospital of Hebei Medical University, No.139, ZiQiang Lu, Shijiazhuang, 050051 Hebei PR China; 3 NHC Key Laboratory of Intelligent Orthopeadic Equipment, The Third Hospital of Hebei Medical University, No.139, ZiQiang Lu, Shijiazhuang, People’s Republic of China

**Keywords:** Acute heart failure, Hip fracture, Risk factors, Prognosis, Elderly

## Abstract

**Background:**

Elderly patients with hip fracture who develop perioperative acute heart failure (AHF) have a poor prognosis. The aim of the present study is to investigate the potential risks of AHF in elderly hip-fracture patients in the postoperative period and to evaluate the prognostic significance of AHF.

**Methods:**

A retrospective analysis was conducted on hip fracture patients at the Third Hospital of Hebei Medical University, who were continuously in hospital from September 2018 to August 2020. To identify independent risk factors for AHF in elderly patients with hip fracture, univariate and multivariate Logistic regression analysis was employed. The Kaplan-Meier survival curve illustrated the relationship between all-cause mortality in the AHF and non-AHF groups. An assessment of the correlation between baseline factors and all-cause mortality was conducted by means of univariable and multivariable Cox proportional hazards analysis.

**Results:**

We eventually recruited 492 patients,318 of whom were in the AHF group. Statistical significance was found between the two groups for age group, concomitant coronary heart disease, COPD, haemoglobin level below 100 g/L on admission, albumin level below 40 g/L on admission, and increased intraoperative blood loss. Age over 75, concomitant coronary artery disease, hemoglobin level below 100 g/L and albumin level below 40 g/L on admission were independent risk factors for AHF in older hip fracture patients. The AHF group exhibited a higher incidence of perioperative complications, such as anemia, cardiovascular issues, and stress hyperglycemia, as well as all-cause mortality. Based on our COX regression analysis, we have identified that the main risk factors for all-cause mortality in AHF patients are concomitant coronary heart disease, absence of pulmonary infection, absence of diabetes, absence of cancer, and absence of urinary tract infection.

**Conclusion:**

Enhancing hip fracture prevention for AHF is particularly important. It is crucial to make informed decisions to avoid poor prognoses. Patients whose age over 75 years old, concomitant coronary heart disease, hemoglobin < 100 g/L and album< 40 g/L on admission are more likely to develop perioperative AHF. To avert complications and potential fatalities, patients with AHF must receive appropriate care during the perioperative period.

## Introduction

Heart failure, a condition that is both fatal and widespread among physicians and surgeons, and disproportionately affects the elderly, with one study showing adult HF rates of 6.6 and 10.6% in men aged 60–79 and ≥ 80, and 4.8 and 13.5% in women, respectively [1]. Heart failure can lead to increased postoperative complications, prolonged hospital stay, and increased all-cause mortality [2], and it has also been reported as an important cause of early mortality in patients with hip fracture [3, 4]. Accidental hospitalisation is a common outcome for patients over 65, particularly those with hip fracture, who develop AHF [5]. This condition is often caused by infection, arrhythmia or myocardial ischaemia and is characterised by a sudden fall in cardiac output, a sudden rise in systemic or pulmonary circulatory pressure, tissue peritoneal insufficiency and acute pulmonary congestion [6]. Research has shown that 6–20% of hip fracture patients suffer from acute heart failure before surgery [7]. Jantzen C. et al. found that the most frequent complication among elderly patients with hip fracture was congestive heart failure [8]. You Fei et al. [9] have reported that age ≥ 70 years old, hypertension, anemia, hypoalbuminemia, and operation time of 120 minutes or longer are risk factors for elderly hip fracture complicated with postoperative heart failure. Identifying and controlling these predisposing and risk factors is necessary to understand and prevent their occurrence and development. Moreover, intervening in the preventable risk factors will allow clinicians to achieve the most favorable outcome for patients with hip fracture.

Due to economic development and advancements in medical care, the aging population in China has become a significant concern, resulting in an increased risk of hip fractures [10]. Globally, 1.7 million hip fractures take place annually, and it is projected that this number will rise to over 6 million by 2050 [11]. Hip fracture is a common type of fracture, which can increase morbidity and mortality [12],lead to poor prognosis, and impose a heavy burden on families and society [13]. The cumulative mortality rate for patients with a hip fracture was 30% at 1 year and 40% at 3 years, according to Malafarina and Guzon-Illescas [14, 15]. In a long-term survey of postoperative complications in elderly patients with hip fractures from 2000 to 2019, Yu Jiang et al. discovered that the incidence of complications increased significantly in patients with pre-existing conditions such as hypertension, type 2 diabetes, coronary heart disease, stroke, arrhythmia, myocardial infarction, and tumors [16].

To our konwlwdge, there have been more studies on the prognosis of heart failure after hip fracture [17, 18]. The short-term postponement of surgery due to perioperative AHF can lead to prolonged bed rest, and can result in serious complications, such as lung infection and deep venous thrombosis of the lower extremities, for those who have broken a hip. Due to pain, anxiety and other factors, elderly patients with hip fracture are susceptible to traumatic stress reaction [19],which increases the risk of complications. It is important to provide these patients with the attention they deserve. However, little research has been done on the prognosis of patients with AHF after hip fracture.

This report sought (1) to clarify the risk factors that could influence perioperative AHF in such patients; (2) to explore the impact of perioperative AHF on prognosis in elderly patients with hip fractures; and (3) to investigate the predictors of all-cause death in perioperative AHF patients.

## Methods

### Patients and groups

A retrospective analysis of hip fracture patients in the Third Hospital of Hebei Medical University’s Department of Orthopedics, who were hospitalized from September 2018 to August 2020, was conducted. The research program received approval from the review committee and was granted exemption from informed consent by the Third Hospital. Those aged 65 and over, with a hip fracture complicated by acute heart failure, a low energy injury (falling from a height), and who are willing to participate voluntarily, as well as having normal communication and understanding abilities, are included in this study. Conversely, those with previously diagnosed heart failure, pathological fracture or old fracture, incomplete clinical data, and those who are unwilling to participate are excluded. In compliance with European Society of Cardiology (ESC) guidelines [20], two groups were identified for the study:(1) individuals with acute heart failure (AHF) and (2) individuals without AHF (Non-AHF). All patients with AHF were diagnosed accordingly.

### Data collection

Demographic characteristics such as age and sex, comorbidities, laboratory indicators such as Hb, CRP, and albumin on admission, and prognostic indicators such as perioperative complications, length of stay, and all-cause mortality were collected. Other indicators such as type of hip fracture, perioperative waiting time, type of anaesthesia, and type of surgery were also recorded. A follow-up period was conducted from discharge to August 31, 2022, during which the patients’ deaths were notified to their relatives via phone calls.

### Definition

Clinical practice has shown that acute heart failure in elderly patients often presents in an atypical manner. Therefore, our diagnosis of AHF is primarily based on BNP concentration. Patients are deemed AHF if their BNP levels increase during hospitalization, even if they were not high upon admission. Additionally, patients may be also diagnosed with AHF if they have elevated BNP levels upon admission but no prior history of heart failure is noted, or if acute heart failure is documented in the medical record.

### Statistical analysis

The Kolmogorov-Smirnov test was employed to assess the normal distribution, the independent t test to evaluate the differences between groups, and the Mann-Whitney U test to analyze non-normally distributed variables. Variables that are continuous are expressed as either mean ± standard deviation (SD) or median (IQR), depending on the distribution. Frequency (percentage) is used to compare categorical variables, which are then expressed in chi-square tests. Kaplan-Meier methods were employed to compare survival rates, while log-rank tests were used to evaluate any disparities. To determine independent risk factors for acute heart failure in elderly patients with hip fractures, univariate and multivariate Logistic regression analysis was conducted. Cox proportional hazards analysis was then utilized to evaluate the correlation between baseline variables and all-cause mortality. Including the factors with a *P*-value of less than 0.2 in the univariate analysis, the Logistic and COX regression models were subjected to multivariate analysis. All statistical analyses were conducted using SPSS software (version 27.0), and a P-value of less than 0.05 was deemed statistically significant.

## Results

### Demographic characteristics

**Fig. 1:**
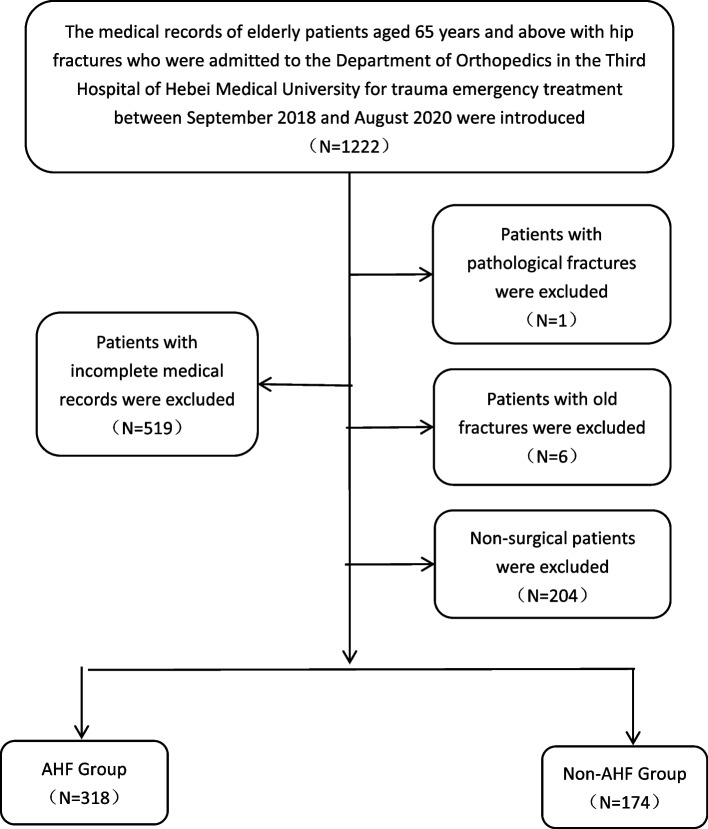
The fow diagram of this study

A total of 1222 patients were admitted to our department for hip fracture between September 2018 and August 2020, and patients with incomplete data and non-surgical treatment were excluded. Finally, 492 patients were included in the study (Fig. 1). The baseline characteristics of the 492 patients, with females making up the majority at 70.1%, are presented in Table 1 and their mean age was 80 years (IQR, 73–85). The largest proportion of these participants were aged over 75 (70.5%).Table 1 revealed no statistically relevant distinctions between the two groups in terms of gender, comorbidities (hypertension, stroke, diabetes, COPD, cancer), fracture type, fracture site, admission within 48 hours of injury, CRP being higher than 5 mg/dl upon admission, anesthesia technique, surgery type, and preoperative waiting period. Significant differences were observed in age, age group, coronary heart disease, arrhythmia, hemoglobin levels and albumin levels upon admission, as well as intraoperative blood loss (*P* < 0.20) at admission.

**Table 1:** Baseline characteristics of patients with or without AHF in old patients with hip fracture

Varibles	Total(492)	AHF Group(318)	Non-AHF Group(174)	*P* value
Age (IQR),years	80(73–85)	81(76–86)	76(71–83)	**< 0.001**
Age group,n(%)				**< 0.001**
< 75 years	145(29.5)	68(21.4)	77(44.3)	
> = 75 years	347(70.5)	250(78.6)	97(55.7)	
Gender,n(%)				0.835
Males	147(29.9)	94(29.6)	53(30.5)	
Female	345(70.1)	224(70.4)	121(69.5)	
Comodity,n(%)				
Hypertension	243(49.4)	157(49.4)	86(49.4)	0.991
Stroke	267(54.3)	171(53.8)	96(55.2)	0.766
Conoary artery diease	146(29.7)	83(26.1)	63(36.2)	**0.019**
Diabetes	115(23.4)	76(23.9)	39(22.4)	0.710
COPD	11(2.2)	9(2.8)	2(1.1)	0.228
Arrhythmology	20(4.1)	10(3.1)	10(5.7)	**0.162**
Cancer	20(4.1)	14(4.4)	6(3.4)	0.608
Fracture type,n(%)				0.626
Femoral neck	267(54.3)	170(53.5)	97(55.7)	
Intertrochanteric	225(45.7)	148(46.5)	77(44.3)	
Fracture site,n(%)				0.843
Left	260(52.8)	167(52.5)	93(53.4)	
Right	232(47.2)	151(47.5)	81(46.6)	
The time from injury to hospital admission < 48 hours,n(%)	378(76.8)	245(77.0)	133(76.4)	0.879
Laboratory index				
Admission Hb,n(%)				**< 0.001**
< 10 g/L	105(21.3)	84(26.4)	21(12.1)	
> = 10 g/L	387(78.7)	234(73.6)	153(87.9)	
Admission Alb,n(%)				**< 0.001**
< 40 g/L	381(77.4)	262(82.4)	119(68.4)	
> = 40 g/L	111(22.6)	56(17.6)	55(31.6)	
Admission CRP,n(%)				0.882
<=5 mg/L	100(20.3)	64(20.1)	36(20.7)	
> 5 mg/L	392(79.7)	254(79.9)	138(79.3)	
Anesthesia,n(%)				0.587
General	226(45.9)	141(44.3)	85(48.9)	
Regional	227(46.1)	150(47.2)	77(44.3)	
Both	39(7.9)	27(8.5)	12(6.9)	
Surgical type,n(%)				0.674
Hip replacement	241(49.0)	158(49.7)	83(47.7)	
Closed reduction and internal fixation	251(51.0)	160(50.3)	91(52.3)	
Preoperative waiting time (IQR),days	4(3–6)	4(3–6)	5(3–6)	0.843
Intraoperative blood loss (IQR),ml	200(200–300)	200(200–300)	215(200–300)	**0.083**

### Clinical variables predicting the appearance of AHF

The relationship between clinical variables associated with AHF in elderly patients with hip fracture is shown in Table 2. In old patients with hip fracture, over 75 years old, combined coronary heart disease, combined arrhythmology, hemoglobin < 100 g/L at admission, albumin < 40 g/L at admission, and increased intraoperative blood loss were associated with the occurrence of AHF according to univariate Logistic analysis (*p* < 0.20). These factors were then incorporated into the multivariate logistic proportional hazards model. Elderly patients with hip fracture who are over 75 years old (HR 2.691, 95% CI 1.764–4.104), have combined coronary heart disease (HR 1.837, 95% CI 1.203–2.806), hemoglobin levels below 100 g/L at admission (HR 2.060, 95% CI 1.190–3.566), and albumin levels below 40 g/L at admission (HR 1.625, 95% CI 1.022–2.584) are at an increased risk for perioperative AHF.

**Table 2:** Univariate and multivariate Logistic regression analysis for factors associated with perioperative AHF in old patients with hip fracture

Varibles	Univariate		Multivariate	
	HR(95% CI)	*P* value	HR(95% CI)	*P* value
Age > =75 years	2.918(1.953–4.361)	< 0.001	2.691(1.764–4.104)	**< 0.001**
Conoary artery diease	1.607(1.080–2.392)	0.019	1.837(1.203–2.806)	**0.005**
Arrhythmology	1.878(0.766–4.604)	0.168	2.052(0.797–5.283)	0.136
Admission Hb < 100 g/L	2.615(1.555–4.398)	< 0.001	2.060(1.190–3.566)	**0.010**
Admission Alb< 40 g/L	2.162(1.406–3.326)	< 0.001	1.625(1.022–2.584)	**0.040**
Intraoperative blood loss	0.999(0.998–1.000)	0.048	0.999(0.998–1.000)	0.088

### Prognosis

**Fig. 2:**
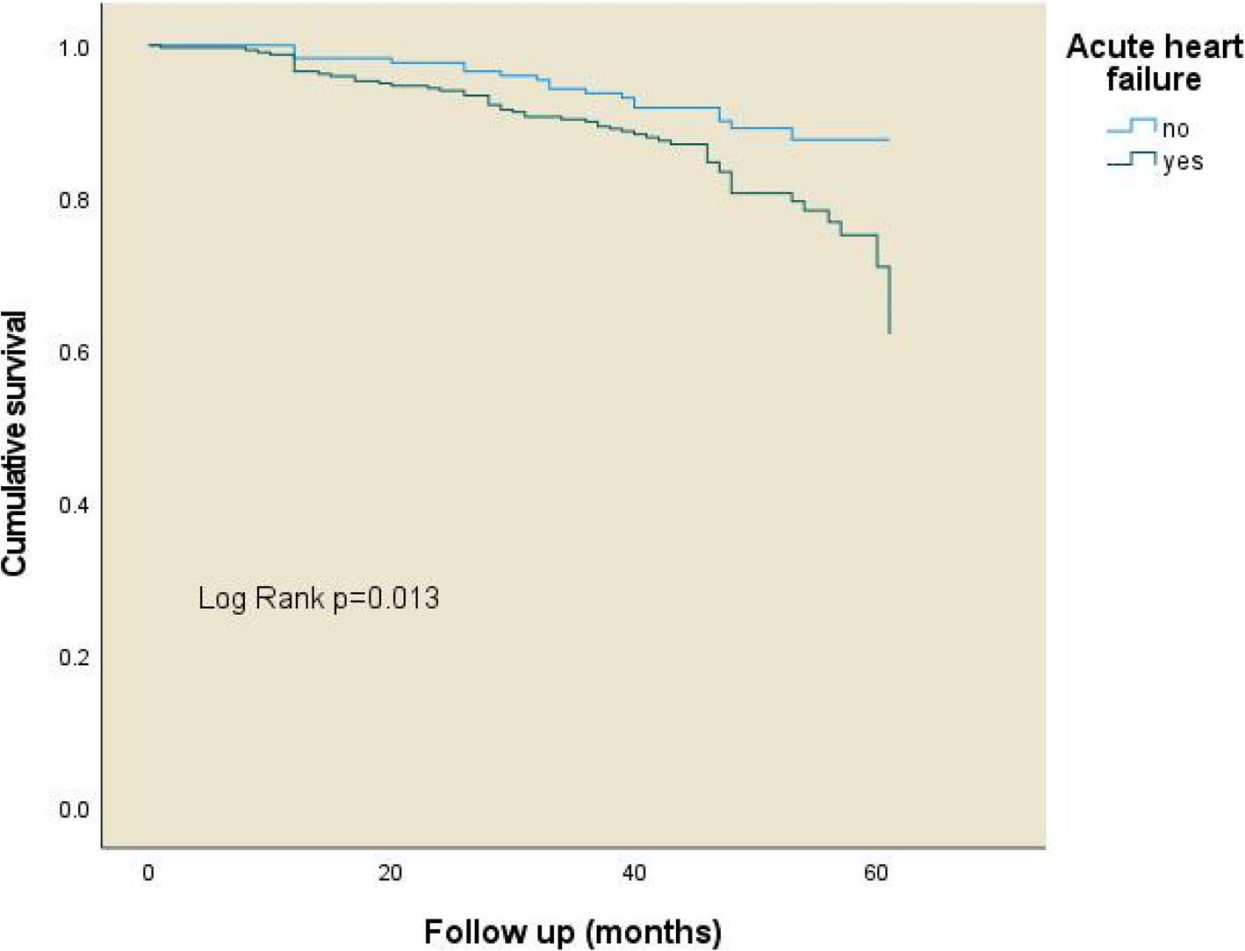
The Kaplan-Meier curve for perioperative acute heart failure

Table 3 reveals the consequences of perioperative complications, length of stay, and all-cause mortality in elderly hip fracture patients with or without perioperative AHF. The incidence of adverse cardiovascular events, stress hyperglycemia, and anemia was higher in those with AHF, which was statistically significant (*P* < 0.05). However, there was no significant difference in the length of stay between the two groups. We also found that hypoproteinemia was the most common complication in both groups. At the end of the research, 56 fatalities (17.6%) were recorded in the AHF cohort, which was higher than the non-AHF group (10.3%), as demonstrated in the Kaplan-Meier survival curve (Fig. 2) (Log Rank *p* = 0.013).

**Table 3:** Comparison of the outcome of patients with or without AHF in old patients with hip fracture

Varibles	AHF(318)	Non-AHF(174)	*P*-value
Perioperative complications,n(%)			
Deep vein thrombosis	151(47.5)	87(50.0)	0.593
Pulmonary infection	161(46.0)	80(46.0)	0.324
Adverse cardiovascular events	127(39.9)	53(30.5)	**0.037**
Acute cerebrovascular disease	31(9.7)	12(6.9)	0.284
Stress hyperglycemia	54(17.0)	11(6.3)	**< 0.001**
Stress ulcer	4(1.3)	5(2.9)	0.354
Urinary tract infection	55(17.3)	30(17.2)	0.988
Anaemia	244(76.7)	116(66.7)	**0.016**
Hypoproteinemia	275(86.5)	145(83.3)	0.345
Electrolyte disturbance	225(70.8)	114(65.5)	0.230
Hospital stay (IQR),days	12(10–16)	12.5(9–16)	0.901
All-cause morlitay	56(17.6)	18(10.3)	**0.031**

### Clinical variables predicting all-cause mortality of AHF

**Fig. 3:**
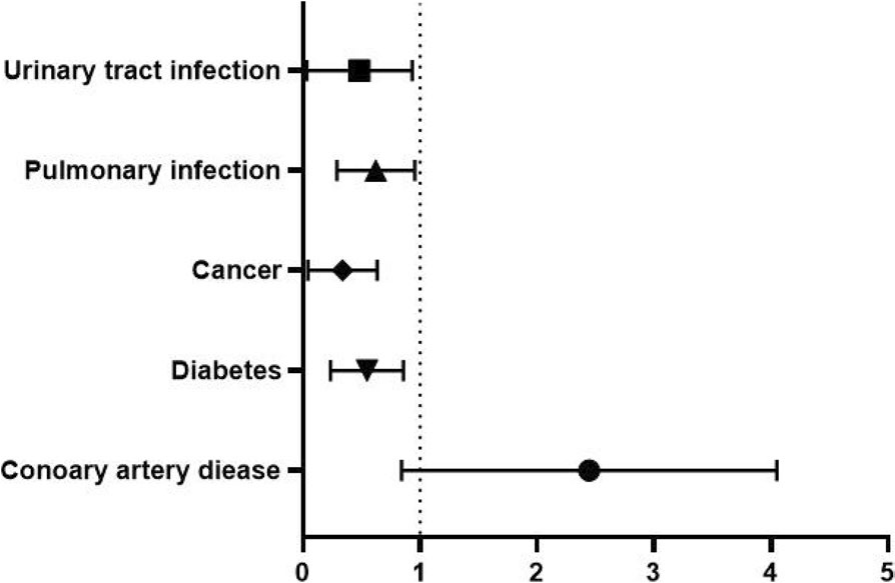
The forest map of risk factors for all-cause mortality in patients with AHF

Table 4 describes the relationship between all-cause mortality and pertinent clinical variables in elderly patients with perioperative hip fracture AHF. An analysis of univariate COX revealed a correlation between all-cause death in patients with AHF and coronary heart disease, no prior comorbidities (cancer, COPD, diabetes), fracture type, time from injury to hospital admission < 48 h, no related complications (deep vein thrombosis, pulmonary infection, urinary tract infection). Additionally, longer preoperative waiting time, longer operation time, and longer hospital stay were also found to be correlated with all-cause death (*p* < 0.20). The multivariate Logistic proportional risk model incorporated the factors chosen by the univariate COX regression analysis. The forest plot in Fig. 3 reveals the independent risk factors for all-cause mortality in patients with AHF, which include coronary heart disease (HR 2.096,95%CI 1.046–4.197), absence of diabetes (HR 0.487,95%CI 0.268–0.884), cancer (HR 0.252,95%CI 0.095–0.669), no pulmonary infection (HR 0.565,95%CI 0.322–0.981) and no urinary tract infection (HR 0.341,95%CI 0.118–0.988). Figure 3 presents the risk factors that predict all-cause mortality in patients with AHF using a forest plot.

**Table 4:** Univariate and multivariate COX regression analysis of all-cause mortality associated with perioperative AHF in elderly patients with hip fracture

Varibles	Univariate Analysis HR (95% CI)	*P* value	Multivariate Analysis HR (95% CI)	*P* value
Age > =75 years	1.343(0.658–2.742)	0.418		
Gender	1.042(0.576–1.885)	0.892		
Comodity				
Hypertension	0.771(0.454–1.307)	0.334		
Stroke	1.206(0.712–2.043)	0.485		
Conoary artery diease	1.611(0.832–3.118)	**0.157**	2.096(1.046–4.197)	**0.037**
Diabetes	0.598(0.340–1.052)	**0.075**	0.487(0.268–0.884)	**0.018**
COPD	0.342(0.106–1.104)	**0.073**	0.509(0.151–1.719)	0.277
Arrhythmology	1.930(0.266–13.990)	0.515		
Cancer	0.323(0.128–0.816)	**0.017**	0.252(0.095–0.669)	**0.006**
Fracture type	1.518(0.893–2.578)	**0.123**	1.470(0.827–2.614)	0.190
Fracture site	0.790(0.465–1.343)	0.384		
The time from injury to hospital admission < 48 hours	1.498(0.838–2.677)	**0.173**	1.786(0.967–3.297)	0.064
Laboratory index				
Admission Hb < 100 g/L	1.205(0.656–2.213)	0.547		
Admission Alb< 40 g/L	0.813(0.384–1.721)	0.588		
Admission CRP > 5 mg/dL	0.940(0.438–2.016)	0.873		
Perioperative complications				
Deep vein thrombosis	0.685(0.404–1.161)	**0.160**	0.714(0.414–1.230)	0.225
Pulmonary infection	0.615(0.358–1.056)	**0.078**	0.565(0.322–0.981)	**0.046**
Adverse cardiovascular events	0.746(0.429–1.298)	0.300		
Acute cerebrovascular disease	1.109(0.475–2.589)	0.812		
Stress hyperglycemia	1.434(0.770–2.671)	0.255		
Stress ulcer	1.850(0.255–13.413)	0.543		
Urinary tract infection	0.467(0.168–1.300)	**0.145**	0.341(0.118–0.988)	**0.047**
Anaemia	1.234(0.649–2.345)	0.522		
Hypoproteinemia	0.758(0.356–1.612)	0.472		
Electrolyte disturbance	0.926(0.528–1.623)	0.788		
Hospital stay	1.030(0.986–1.076)	**0.180**	1.020(0.954–1.089)	0.567
Anesthesia	0.972(0.864–1.093)	0.632		
Surgical type	1.391(0.816–2.370)	0.225		
Preoperative waiting time	1.053(0.982–1.130)	**0.147**	1.015(0.909–1.133)	0.791
Intraoperative blood loss	1.000(0.998–1.002)	0.834		
Operation time	0.994(0.986–1.002)	**0.125**	0.994(0.986–1.002)	0.147

## Discussion

A meta-analysis revealed that those with heart failure had a greater likelihood of hip fracture than those without [21]. With regards to prognosis, it has been demonstrated that heart failure is a risk factor for both early and late outcomes in patients with hip fractures [22]. In this study, 492hip fractures, including 318 patients(64.6%) with acute heart failure, were included to explore the risk factors and prognosis. It was discovered that women and stroke patients had a higher incidence of hip fractures at an older age with acute heart failure. The common age of AHF patients with hip fracture was over 75 years old. Hypoproteinemia accounted for the highest proportion of perioperative complications. Independent risk factors for perioperative AHF included over 75 years old, combined coronary heart disease, hemoglobin < 10 g/L and album < 40 g/L on admission. The mortality rate of those in the AHF group was significantly greater than that of those in the non-AHF group. Moreover, complications, adverse cardiovascular events, stress hyperglycemia, and anemia were all notably higher in the AHF group.

The results of Ahluwalia et al. [23] were corroborated by multivariate Logistic analysis, which revealed that older age was a risk factor in our study, which largely included AHF patients aged over 75 and some studies that have indicated a greater incidence of heart failure in those aged over 75 than those aged 65 and above [24]. As age increases, fractures such as osteoporotic and frality fractures, particularly hip fractures, become more frequent in the elderly, leading to a decrease in mobility and quality of life [2, 25]. Furthermore, the elderly are vulnerable to a variety of comorbidities, which can lead to a poor prognosis and even hasten death. Although surgery is currently the primary treatment for hip fractures, it is also important to consider the tolerance of the elderly to surgery and their overall physical condition [26].

Among patients with hip fracture, Hb levels below 100 g/L accounted for 21.3%,and some studies have used a cut-off value of less than 100 g/L to determine low Hb levels at admission, so we chose Hb levels below 100 g/L as the cut-off [27, 28]..Looker AC et al. A non-linear association between hemoglobin and the risk of hip fracture was discovered [29]. The mechanism by which low hemoglobin leads to the adverse consequences of fracture may be insufficient oxygen delivery to the tissue [30]. The main source of anemia due to hip fracture, particularly intertrochanteric fracture of the femur, is an overabundance of blood, which then leads to a lack of appetite and myocardial ischemia. Low hemoglobin levels can cause a heightened rate of re-hospitalization and other cardiovascular issues in those with AHF [31]. Anemia caused by low hemoglobin can aggravate heart failure decompensation and increase mortality through salt and water retention, decreased renal blood flow and glomerular filtration rate, and neurohormone activation [32]. Complications, readmission rates, and extended hospital stays may all be factors in the rise of mortality; albumin, a measure of nutrition, being a poor indicator of such, is a sign of inadequate nutrition. The common causes of low protein are low albumin intake and reduced absorption in the elderly, and long-term bed rest after fracture will slow down gastrointestinal motility, which will aggravate this phenomenon. Low albumin will lead to poorer wound healing, resulting in prolonged bed rest, resulting in various complications, and even an increase in mortality [33]. Greenhalgh et al. concluded through a retrospective cohort study that a hemoglobin of 80 g/L was the threshold for blood transfusion in patients with hip fractures [34]. However, since the elderly are a special group, our hospital believed that blood transfusion was necessary if the hemoglobin was less than 100 g/L.When the patient’s albumin is less than 30 g/L, we will administer the measure of albumin infusion. However, medicine is not as good as food, and we will do our best to persuade patients to supplement their nutrition through oral intake.

It was determined that elderly patients with hip fracture were more likely to suffer from AHF when combined with coronary artery disease (CAD). Zheng J et al. believed that coronary artery disease is an important independent risk factor for perioperative AHF in elderly patients with hip fracture [35], consistent with our findings. To our knowledge, coronary artery disease is the cause of myocardial ischemia, resulting in myocardial hypoperfusion. This sustained hypoperfusion leads to reduced oxygen delivery and sympathetic excitation, in which case a surge of catecholamines can affect surviving muscle cells and impair myocardial contractility [36]. Myocardial damage can be the result of hibernating or depressed myocardium, which is a common symptom of acute heart failure complicated by coronary heart disease in patients. Studies have shown that the diagnosis of AHF with CAD is associated with a poor prognosis [37]. The link between hip fracture and coronary heart disease has been explored in the past, leading to the conclusion that coronary artery disease is a potential cause of hip fracture [38]. Coronary artery calcification, endothelial dysfunction, and inflammation may be the pathophysiological factors that lead to coronary artery disease and hip fracture [39].CAD is a result of numerous elements and necessitates multiple system issues that should be taken into account. In acute heart failure, subendocardial ischemia may result from an increase in the left ventricular end-diastolic pressure. Over-activation of neurohormones may exacerbate myocardial ischemia by increasing myocardial contractility and decreasing coronary perfusion due to endothelial dysfunction [40], thus leading to adverse cardiovascular events. The significant increase in catecholamine induced by sympathetic nerve stimulation, is a critical factor in the pathophysiology, as evidenced by CRP in clinical laboratory tests - which is in agreement with the article’s findings that the AHF group has a higher CRP than the non-AHF group [41]. For patients with coronary heart disease and without hypotension, we will routinely give vasodilator drugs and ECG monitoring after admission. Markers such as troponin were also monitored for further myocardial damage.

Acute heart failure can lead to increased gluconogenesis and insulin resistance through activation of the sympathetic nervous system and excessive release of anti-regulatory hormones and anti-inflammatory cytokines, inducing stress hyperglycemia [42],which is typically observed after hospitalization or within 3 days of surgery. Hyperglycemia at hospitalization is associated with increased all-cause mortality within 1 year in patients with AHF [43]. Hyperglycemia of stress can cause microvascular dysfunction, proinflammatory behavior, and pre-thrombotic state, resulting in acute myocardial infarction and a bleak prognosis [43]. Since this complication is a transient reaction, the stress response can be improved through analgesia and other means, and hypoglycemic drugs should not be overused to avoid the occurrence of hypoglycemia [44].

Patients with AHF have a greater incidence of short-term events and a greater proportion of heart failure deaths than those with chronic heart failure [45]. The causes and processes of death in AHF patients are multifaceted. The severity of heart failure is a major factor, and other elements such as comorbidities, the intensity and duration of hemodynamic overload, and the extent of end-organ damage during the AHF episode may all lead to unfavourable outcomes in this population [46]. According to the risk factors we summarized, CAD is not only a risk factor for perioperative AHF in elderly patients with hip fracture, but also an increased mortality factor. We should pay great attention to it and give corresponding drugs to improve myocardial remodeling. The reason why urinary tract infections and lung infections do not lead to death may be that the prevention of these complications has been strengthened. Our department is composed of first-class orthopedic surgeons, experienced physicians and a team of nurses who are in place to prevent lung infections through aerosol inhalation. It is uncertain how diabetes and cancer do not raise mortality, and disparities due to limited sample sizes can be taken into account.

### Limitation

It is worth deliberating the restrictions of our research. Firstly, because of the retrospective nature of the study, we cannot judge its overall characteristics based on data collected in one hospital alone. Secondly, in some cases, there were differences in some results due to the small sample size.

## Conclusion

A high rate of acute heart failure was observed in elderly patients aged over 75 years in our research. Over 75 years old, combined coronary heart disease, hemoglobin < 10 g/L and album < 40 g/L on admission were independent risk factors for perioperative AHF in elderly patients with hip fracture. The AHF group exhibited a greater prevalence of perioperative complications such as anemia, adverse cardiovascular events, and stress hyperglycemia, with mortality rates significantly higher than those of the non-AHF group. Moreover, we found that combined CAD was also a risk factor for all-cause mortality. Prevention and early detection of AHF are therefore of paramount importance, with CAD management being a key component.

## Data Availability

The data used to support the findings of this study are available from Zhi [1] qian Wang upon request.

## Abbreviations

AHF: Acute heart failure; CAD: Coronary artery disease
Hb: Hemoglobin
Alb: Album
COPD: Chronic obstructive pulmonary disease
